# Editorial: The role of glial cells in the autoimmune diseases of nervous system

**DOI:** 10.3389/fncel.2023.1141622

**Published:** 2023-02-06

**Authors:** Wanwan Min, Hongliang Zhang, Jie Zhu, Mingqin Zhu

**Affiliations:** ^1^Department of Neurology, Neuroscience Center, The First Hospital of Jilin University, Changchun, China; ^2^Department of Life Sciences, National Natural Science Foundation of China, Beijing, China; ^3^Division of Neurogeriatrics, Department of Neurobiology, Care Sciences and Society, Karolinska Institutet, Karolinska University Hospital Solna, Stockholm, Sweden

**Keywords:** autoimmune diseases, microglia, astrocytes, central nervous system, ferroptosis, Piezo1

Glial cells are large type of cells in neural tissue and are widely distributed in the central and peripheral nerves. There is growing evidence that glial cells are actively involved in many complex functions within the central nervous system (CNS) beyond connecting and supporting various neural components, such as immune monitoring and inflammatory response, metabolism and synaptic homeostasis, and blood–brain barrier regulation (Jessen, 2004). Central inflammation is found in most CNS diseases. The inflammatory response of the CNS results from the combined action of all glial cells (Yang and Zhou, 2019). Particularly, microglia, the brain's main innate immune cells, are often the first effector cells stimulated by injury and disease. In recent years, the interaction between glial cells and autoimmune diseases has attracted widespread interest due to the special function of glial cells. The collection of articles in this Research Topic provides deeper insights into the role of glial cells in autoimmune diseases.

Ferroptosis, which is cell death driven by iron-dependent phospholipid peroxidation, is involved in the pathophysiology of many neurological disorders (Yang and Stockwell, 2016; Wang et al., 2022). However, the specific effects of ferroptosis on neurons and glial cells remain unclear. Jiao et al. found that primary cortical astrocytes, microglia, and neurons differ in terms of sensitivity to ferroptosis. Microglia cells were the most sensitive to ferroptosis, and neurons were relatively insensitive. The differences may be explained by the differentially regulated iron metabolism and the ability to handle iron. Microglia may serve as a guardian of ferroptosis within the neuronal system, and the authors found that the cells were more resistant to ferroptosis in the tri-culture system than in the monoculture. Microglia underwent ferroptotic cell death at concentrations where other cells remained intact. Elucidating the cell death patterns in neuron and glial cells in disease settings will provide a theoretical basis for strategies to inhibit brain cell death.

Mechanical damages are also one of the important factors driving the progression of many pathophysiological states. Piezo1, one of the first discovered mechanoreceptors, was found to have physiological functions such as axonal growth regulation or astrocyte reactivity. Velasco-Estevez et al. found that Piezo1 can be expressed in oligodendrocytes and oligodendrocyte progenitor cells (OPC) and where it decreases with glial maturation. GsMTx4 inhibition of Piezo1 can also lead to increased multiplication and migration of MO3.13 oligodendrocytes. This, in turn, may render a protective effect through Ca^2+^ signaling reduction, calpain and phospholipaseA2 activation, and subsequent myelin lipid degradation. Piezo1 also plays a role in CNS pathophysiology. The authors found that the expression of the mechanoreceptor Piezo1 was lower in the brain of patients with multiple sclerosis than in healthy groups. OPC differentiation is inhibited in rigid matrix, while chronic demyelinating disease can lead to abnormal deposition of extracellular matrix components, increasing the overall hardness of tissues (Segel et al., 2019; Urbanski et al., 2019). It has also been suggested that a decrease in Piezo1 may be a potential pathological mechanism for MS myelination aplasia.

Glial cells play a key role in brain physiology, metabolism, development, and central inflammation (Herculano-Houzel, 2014). This Research Topic also includes a series of reviews highlighting the physiological role of various types of glial cells in the nervous system and their contribution to autoimmune diseases. The review by Li et al. covers the roles of glial cells in neurologic autoimmune disorders and the influence of autoantibodies produced in autoimmune disorders on glial cell functions. In addition to attacking neurons, autoantibodies can also attack glial cells, disrupting homeostasis and exacerbating the inflammatory response. Targeting glial cells may be a new strategy in the treatment of autoimmune diseases.

Enteric glial cells (EGCs) are an important component of the enteric nervous system. In addition to supporting the survival and function of the enteric nervous system, EGCs may also have a variety of immune functions (Wallrapp et al., 2022). The review by Liu and Yang covers the evidence of the potential involvement of EGCs in several immune diseases of the gut, including inflammatory bowel disease, celiac disease, and autoimmune enteropathy. They further discussed key immunological aspects of EGCs that deserve the attention of future research.

The articles included in the current collection add much to our comprehension of the involvement of glia in the pathophysiological mechanisms of different autoimmune diseases. The study of glial cells provides a new direction for the diagnosis and treatment of neuroimmune diseases.

## Author contributions

MZ, HZ, and JZ organized the Research Topic and revised the manuscript. WM wrote the manuscript. All authors contributed to the article and approved the submitted version.

## References

[B1] Herculano-Houzel S. (2014). The glia/neuron ratio: how it varies uniformly across brain structures and species and what that means for brain physiology and evolution. Glia 62, 1377–1391. 10.1002/glia.22683 24807023

[B2] Jessen K. R. (2004). Glial cells. Int. J. Biochem. Cell Biol. 36, 1861–1867. 10.1016/j.biocel.2004.02.023 15203098

[B3] Segel M. Neumann B. Hill M. F. Weber I. P. Viscomi C. Zhao C.. (2019). Niche stiffness underlies the ageing of central nervous system progenitor cells. Nature 573, 130–134. 10.1038/s41586-019-1484-9 31413369 PMC7025879

[B4] Urbanski M. M. Brendel M. B. Melendez-Vasquez C. V. (2019). Acute and chronic demyelinated CNS lesions exhibit opposite elastic properties. Sci. Rep. 9, 999. 10.1038/s41598-018-37745-7 30700777 PMC6354022

[B5] Wallrapp A. Yang D. Chiu I. M. (2022). Enteric glial cells mediate gut immunity and repair. Trends Neurosci. 45, 251–253. 10.1016/j.tins.2021.12.001 34973845

[B6] Wang T. Tomas D. Perera N. D. Cuic B. Luikinga S. Viden A.. (2022). Ferroptosis mediates selective motor neuron death in amyotrophic lateral sclerosis. Cell Death Differ. 29, 1187–1198. 10.1038/s41418-021-00910-z 34857917 PMC9177596

[B7] Yang Q. Q. Zhou J. W. (2019). Neuroinflammation in the central nervous system: symphony of glial cells. Glia 67, 1017–1035. 10.1002/glia.23571 30548343

[B8] Yang W. S. Stockwell B. R. (2016). Ferroptosis: death by lipid peroxidation. Trends Cell Biol. 26, 165–176. 10.1016/j.tcb.2015.10.014 26653790 PMC4764384

